# Statins in COVID-19 Therapy

**DOI:** 10.3390/life11060565

**Published:** 2021-06-16

**Authors:** Justyna Olszewska-Parasiewicz, Łukasz Szarpak, Sylwester Rogula, Aleksandra Gąsecka, Urszula Szymańska, Maria Kwiatkowska, Milosz J. Jaguszewski, Radosław Sierpiński, Artur Zaczyński, Waldemar Wierzba, Dariusz A. Kosior

**Affiliations:** 1 Central Clinical Hospital the Ministry of the Interior and Administration, Wołoska 137, 02-507 Warsaw, Poland; justyna.olszewska@cskmswia.pl (J.O.-P.); u.a.szymanska@gmail.com (U.S.); maria.kwiatkowska@cskmswia.pl (M.K.); artur.zaczynski@cskmswia.pl (A.Z.); waldemar.wierzba@cskmswia.pl (W.W.); Dariusz.Kosior@cskmswia.pl (D.A.K.); 2 Maria Sklodowska-Curie Białystok Oncology Centre, Ogrodowa 12, 15-027 Białystok, Poland; 3 Maria Sklodowska-Curie Medical Academy in Warsaw, Solidarnosci 12, 03-411 Warsaw, Poland; 4 Department of Cardiology, Medical University of Warsaw, Banacha 1a, 02-097 Warsaw, Poland; sylwesterrogula@o2.pl (S.R.); aleksandra.gasecka@wum.edu.pl (A.G.); 5 Department of Cardiology, Medical University of Gdańsk, Dębinki 7, 80-952 Gdańsk, Poland; jamilosz@gmail.com; 6 Faculty of Medicine, Collegium Medicum, Cardinal Stefan Wyszyński University, 01-815 Warsaw, Poland; src.emergency@gmail.com; 7 UHE Satellite Campus in Warsaw, University of Humanities and Economics in Łódź, Felińskego 15, 01-513 Warsaw, Poland; 8 Mossakowski Medical Research Institute, Polish Academy of Sciences, 02-106 Warsaw, Poland

**Keywords:** COVID-19, SARS-CoV-2, pleiotropic effects, statins, therapy

## Abstract

Inhibitors of 3-hydroxy-3methylgultaryl-coenzyme A reductase (statins) are one of the main groups of drugs used in preventing and treating cardiovascular diseases worldwide. They are widely available, cheap, and well-tolerated. Based on statins’ pleiotropic properties, including improvement of endothelial dysfunction, antioxidant properties, atherosclerotic plaque stabilization, and inhibition of inflammatory responses, it can be hypothesized that the use of statins, at least as an adjuvant in antiviral therapy, may be justified. All these effects might be especially beneficial in patients with COVID-19, suffering from endothelial dysfunction, microvascular and macrovascular thrombosis, and cytokine storm. Here, we review the recent data regarding the pathophysiology of SARS-CoV-2 activity in host cells, proposed COVID-19 therapy, the pleiotropic activity of statins, and statins in clinical trials in respiratory infections. According to the guidelines of the European and American Cardiac Societies, in patients with cardiovascular disease or high cardiovascular risk with concomitant COVID-19 it is recommended to continue statin treatment. However, the initiation of statin therapy de novo in COVID-19 treatment should only be done as part of a clinical trial.

## 1. Introduction

Pneumonia is the main manifestation of SARS-CoV-2 infection, which may lead to severe respiratory failure. Due to the cardiotropic potential of the virus, the cardiovascular system is involved in a high percentage of cases [1]. It has been observed that patients with COVID-19, who also have cardiovascular risk factors, such as male gender, older age, diabetes, arterial hypertension, and obesity, as well as patients with pre-existing cardiovascular disease, are especially prone to increased morbidity, more severe and rapid cases of COVID-19, and thus also increased mortality [2].

In a significant proportion of patients, cardiac injury may occur due to COVID-19 infection [3]. COVID-19 is associated with enhanced inflammation (the so-called cytokine storm), abnormal activation of the coagulation system, and decompensation of the cardiovascular system [4]. Except for thromboembolic complications such as acute coronary syndromes and venous thromboembolism, myocarditis also plays an important role in the development of acute heart failure in patients with COVID-19 [5]. In autopsy reports, SARS-CoV-2 RNA was found in cardiomyocytes of 60% of patients with a severe case of the disease; however, symptomatic myocarditis occurred only in 10% of cases [6]. In almost 40% of patients with severe SARS-CoV-2 infection there is an increase in plasma concentration of biomarkers of cardiac injury and overload (highly sensitive cardiac troponin I–hs cTnI; N-terminal pro B-type natriuretic peptide–NT-proBNP), which is associated with poor prognosis [3]. It has also been reported that supraventricular and ventricular arrhythmias may complicate the course of COVID-19 [7]. COVID-19 is associated with hemodynamic decompensation and hypoxemia caused by gas exchange failure in the lungs, but also proarrhythmic side effects of COVID-19 treatment (antiviral agents and antibiotics, e.g., ritonavir, or azithromycin) [8].

In patients with confirmed COVID-19 infection, the optimal treatment of cardiovascular diseases, prevention of their decompensation, and prevention of heart and vessel damage is as important as antiviral therapy. Unfortunately, there is no convincing evidence for the efficacy of any medication in SARS-CoV-2 treatment. The reports so far indicate the possibility of implementing treatment with convalescent plasma, tocilizumab, remdesivir, lopinavir/ritonavir or favipiravir [9,10,11,12]. Due to the current lack of reliable confirmation of the efficacy of proposed therapies and their potential side effects, it is recommended to use them only in hospital settings as part of clinical trials [13]. In the absence of a specific treatment, it is crucial to establish the effect of co-administered medications in patients with COVID-19. Among cardiovascular medications, statins play a crucial role both in primary and secondary prevention of cardiovascular events [14]. Moreover, statins have well-established pleiotropic effects, including improvement of endothelial dysfunction, antioxidant properties, atherosclerotic plaque stabilization, and inhibition of inflammatory responses [14]. All these effects might be especially beneficial in patients with COVID-19 suffering from endothelial dysfunction, microvascular, and macrovascular thrombosis, and cytokine storms [15,16]. Here, we review the pathophysiology of SARS-CoV-2 activity in host cells, proposed COVID-19 therapy, the pleiotropic activity of statins, and statins in clinical trials in respiratory infections.

## 2. Pathophysiology of SARS-CoV-2 Activity in Host Cells

The increasing number of clinical data suggest that endothelium dysfunction plays a significant role in the pathophysiology of COVID-19 disease [17,18]. SARS-CoV-2 infection leads to an enhanced immune response caused by the entry of the virus into the cell by affecting the expression of angiotensin converting enzyme 2 (ACE2) receptor, by inhibiting Toll-like receptors (TLR)–myeloid differentiation primary response 88 (MyD88)–nuclear factor-κB (NF-B) signaling pathway, and the development of cytokine storm with the participation of interleukin 6 (IL-6) [17]. Blocking one of those signaling pathway mechanisms might be a new, promising therapeutic approach for patients with COVID-19.

The currently postulated mechanism for SARS-Cov-2 entry into the host cells is binding SARS-CoV-2 spike proteins with ACE2 receptors [19]. ACE2 distribution and expression might be critical for target organs of SARS-CoV-2 infection (especially lungs, vessels, heart, and kidneys). One of the main roles of ACE2 is the hydrolysis of angiotensin II (Ang-2) to angiotensin 1–7 (Ang 1–7). Ang-2 binding to AT1 receptors activates the renin-angiotensin-aldosterone system, which leads to vasoconstriction (hypertension), the development of chronic inflammation (including organ fibrosis), and thrombosis [20]. SARS-CoV-2 entering into cells reduces the expression of ACE2 receptors (down-regulation), and is associated with the loss of catalytic activity of these membrane receptors. Clinical reports regarding patients with SARS-CoV-2 infection reveal that the ACE2 down-regulation induced by virus invasion might be especially detrimental in humans with pre-existing ACE2 deficiency, which is observed in elderly patients with hypertension, diabetes, and other cardiovascular diseases. This group is therefore particularly exposed to severe cases of COVID-19 and its complications [21].

It has been proven in a mouse model that the TLR-MyD88-NF-κB signaling pathway physiologically stabilizes the production of proinflammatory cytokines (especially IL-1, 6,8,18), which allows the maintenance of the integrity and function of the endothelium and prevents cytokine storm. In SARS-CoV-2 infection, expression of MyD88 gene (Toll-like activity regulator TLR) is impaired and thus the enhanced NF-κB protein is produced. Clinically, it worsens the course of pneumonia, which may lead to the development of sepsis and multiple organ failure [22].

Some studies suggest that dyslipidemia occurs in COVID-19 patients. A study including 597 COVID-19 patients (mild: 394; severe: 171; critical: 32) has shown that LDL levels were significantly lower in COVID-19 patients as compared with normal subjects (*p* < 0.001) [23]. Furthermore, LDL levels are associated with severity and mortality of the disease [24]. The mechanism underlying LDL lowering in patients suffering from SARS-CoV-2 infection is complex. First, it may result from a liver injury due to COVID-19. Second, viral infection induces the release of pro-inflammatory cytokines, which modulate lipid metabolism including oxidation of LDL by reactive oxygen species, thus facilitating LDL clearance [25]. Third, COVID-19 patients may have an increased vascular permeability caused by the virus-induced inflammation. Exudative fluids contain high levels of protein and cholesterol, leading to their evacuation from the bloodstream [26].

## 3. Proposed COVID-19 Therapies

**Figure 1:**
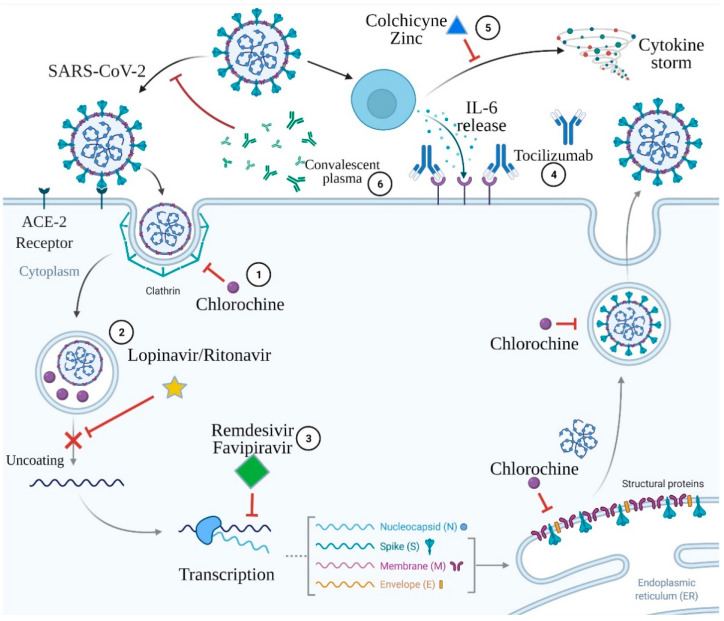
Mechanisms of action of the proposed COVID-19 therapies. 1. Chloroquine acts via deprivation of formation of the clathrin complexes during SARS-CoV-2 endocytosis and via interruption of post-translation modification of the virus proteins. 2. Lopinavir/ritonavir inhibits virus protease and subsequently impairs virus uncoating. 3. Remdesivir and favipiravir are both antimetabolites that deprivate RNA polymerase function, which results in the premature termination of viral RNA transcription. 4. Tocilizumab is a monoclonal antibody against the interleukin-6 receptor. 5. Colchicine and zinc prevent cytokine storm development. 6. Convalescent plasma contains antibodies capable of binding to and destroying the virus. Created with BioRender.com.

The proposed COVID-19 therapies include convalescent plasma, tocilizumab, remdesivir, lopinavir/ritonavir, favipiravir or chloroquine and hydroxychloroquine, colchicine, and zinc supplementation. The mechanisms of action of the proposed COVID-19 therapies above are presented in Figure 1.

Since the end of 19th century, passive immune therapy has been successfully used to treat infectious diseases [27]. Prior to the availability of monoclonal antibodies and gamma globulin products, passive antibody therapy relied on the use of convalescent plasma or serum. Those immune blood products were collected from the recovered donors or animals as therapeutic agents for at-risk or infected patients, both for the purpose of prophylaxis or treatment of a specific pathogen [28]. Vaccination (active immunization therapy) requires an extended period of time to develop an immune response and can display a wide range of clinical variability among recipients [28]. Moreover, the production of a widely available, effective vaccine requires time. In contrast to vaccination, passive antibodies exert their immune activity immediately after administration, which involves the transfer of pre-formed antibodies. Neutralizing antibodies that bind to a pathogen restricts the entry of the pathogen into host cells and enhances clearance of the pathogen via antibody-dependent phagocytosis, antibody-dependent cellular toxicity, and/or complement activation [29]. Hence, convalescent plasma has the potential to confer immediate immunity among at-risk or infected patients, reducing the societal disease burden during large-scale pandemics [28].

Historically, the whole blood, convalescent blood products, antibodies obtained from animals, and more recently, monoclonal or polyclonal antibodies were used as a passive immunotherapy. Plasma collection by apheresis with subsequent convalescent plasma transfusion is the most popular passive immunotherapy strategy during pandemics [30]. Patients who have recovered from an infectious disease have a blood product withdrawn via venipuncture. Then, blood is screened for neutralizing antibodies specific to the causative pathogen. In perfect conditions, high-concentration neutralizing antibody convalescent plasma is used for therapy. There are two possible ways to use convalescent plasma: (i) it may be transfused to non-infected individuals to provide passive immunity to the recipient, or (ii) it can be used to ameliorate the disease course in infected individuals [28,30]. A major principle of convalescent plasma therapy is that the plasma must be given early in the course of the infectious disease to maximize the clinical or mortality benefits [31].

Convalescent plasma has previously been used in the management of coronaviruses (CoVs) [32]. Two major epidemics caused by CoVs occurred in the 21st century: (i) the 2003 SARS-CoV-1 epidemic originating in Hong Kong and (ii) the 2012 MERS-CoV epidemic, which originated in Saudi Arabia. Both were associated with high mortality and lack of therapeutic options. Hence, there was a need for the use of convalescent plasma. A meta-analysis including eight observational studies and 214 patients with SARS demonstrated a mortality benefit following transfusion of convalescent plasma [33].

In the current COVID-19 pandemic, blood collection centers from around the world have established programs for recovered survivors to donate COVID-19 convalescent plasma, which may be obtained from recovered COVID-19 survivors via apheresis or separated from whole blood collected as a standard blood donation. Apheresis collection is strongly preferred. In this process, more units of convalescent plasma per donation are produced. Furthermore, the donor may perform more frequently than standard blood donation [34].

Early work from the current CoV pandemic suggests that SARS-CoV-2 elicits a robust immune response with high levels of antibodies, including immunoglobulins (IgM and IgG), for months after the onset of COVID-19, suggesting a relatively large window of time and high probability of successful extraction of high-titer anti-SARS-CoV-2 plasma [35,36]. There have been several randomized, controlled trials investigating convalescent plasma treatment in patients hospitalized for severe or life-threatening COVID-19 [37,38,39]. Data from all randomized trials and controlled studies shows that convalescent plasma treatment is associated with a significant reduction in mortality (31% in convalescent plasma group versus 19% in control group; odds ratio, 0.5; 95% confidence interval (CI), 0.40 to 0.69; *p* < 0.001) [40]. Administration of convalescent plasma in the first 44 h of hospitalization was associated with the greatest efficacy [34].

Tocilizumab is mainly used for the treatment of rheumatoid arthritis (RA) and systemic juvenile idiopathic arthritis [41,42]. Tocilizumab selectively and competitively binds to soluble IL-6 receptors and blocks the signaling caused by IL-6 [41]. IL-6 is a key cytokine leading to an inflammatory storm that results in gas exchange dysfunction, especially impaired oxygen diffusion, and eventually leads to pulmonary fibrosis and organ failure [43]. Early serology analyses identified higher IL-6 serum levels in patients with severe COVID-19 when compared to patients with mild or moderate disease [44,45]. It has been proposed that elevated IL-6 levels may be associated with greater disease severity and worse clinical outcomes [46]. Tocilizumab, as a treatment targeting IL-6 receptors, has been reported to improve the clinical outcomes of patients with severe COVID-19 [47]. However, in a randomized, double-blind, placebo-controlled trial, in moderately ill and hospitalized patients with COVID-19, tocilizumab was not effective at preventing death [48]. Although basic science suggests a rationale for the administration of tocilizumab to patients with COVID-19, its efficacy has only been seen in observational studies, but not confirmed in randomized trials.

In the United States of America, three SARS-CoV-2 monoclonal antibody therapies have been granted emergency use authorization for the treatment of non-hospitalized patients with mild-to-moderate COVID-19. These are bamlanivimab as a monotherapy, bamlanivimab together with etesevimab, and casirivimab with imdevimab as a combination therapy [49]. Those antibodies are generated against the receptor-binding domain (RBD) of the spike protein. The anti-RBD monoclonal antibodies prevent binding of the spike protein to its receptor, angiotensin-converting enzyme 2, on target host cells. As a result, they can neutralize the ability of the virus to bind and fuse with the target host [49].

Remdesivir is a monophosphoramidate nucleoside prodrug that undergoes intracellular metabolic conversion to its active form–metabolite nucleoside triphosphate [50]. A recent biochemical analysis revealed that in SARS-CoV-2, remdesivir acts via termination of RNA synthesis at three positions after the position where it is incorporated [51]. The premature termination of RNA results in abruption of transcriptional and translational processes needed for the generation of new virions. The resulting antiviral effects of remdesivir have been studied in different cell-based models [50]. In addition, in a phase 3 randomized controlled trial comparing remdesivir and placebo in 1062 patients, remdesivir had beneficial clinical effects in patients who required oxygen supplementation, but not in critically ill patients who required mechanical ventilation [52]. Remdesivir reduced the time to recovery by 31%, which is a relatively modest, but clear therapeutic effect [52]. A meta-analysis of the two available randomized controlled trials demonstrated a statistically significant reduction in mortality in patients with remdesivir treatment (risk ratio [RR], 0.69 [95% CI, 0.49 to 0.99]) [53]. 

Lopinavir is a HIV-1 protease inhibitor combined with another protease inhibitor [54]. Lopinavir is prescribed in combination with low-dose ritonavir [55], in which ritonavir acts as a pharmacokinetic enhancer [56]. Protease plays an important role in the life cycle of SARS-CoV-2, owing to its role in the cleavage of polyproteins and subsequent release of nonstructural proteins. Lopinavir/ritonavir (L/R) combination has been shown to efficiently bind SARS-CoV-2 protease and exert inhibitory activity against SARS-CoV-2 in vitro. However, in a meta-analysis of 3 studies, L/R treatment showed no difference in terms of mortality and progression of the disease to a more severe state. However, L/R therapy decreased the duration of hospitalization in comparison with standard of care (mean difference: −1.466 [−2.403 to −0.529]) [57].

Favipiravir is a purine base analog. It is a selective and potent inhibitor of the RNA-dependent RNA polymerase (RdRp) of RNA viruses. Favipiravir is incorporated into the nascent viral RNA by error prone viral RdRp, which leads to chain termination and viral mutagenesis [58]. In an open-label, parallel-arm, multicenter, phase 3 trial, 150 patients were randomized into favipiravir (*n* = 75) or placebo (*n* = 75) groups. Among moderately ill patients, median time to clinical cure was 3.5 days (95% CI: 3 days, 4 days) for favipiravir, compared with 6 days (95% CI: 4 days, 12 days) for control (*p* = 0.030). Median time to hospital discharge was 4 days shorter in the moderate subgroup treated with favipiravir (median: 9 days, 95% CI: 6 days, 11 days) when compared with control (median: 13 days, 95% CI: 8 days, 17 days, *p* = 0.067). Based on the significant improvement in time to clinical cure, the authors suggested that favipiravir may be beneficial in mild-to-moderate COVID-19 [59].

Chloroquine and hydroxychloroquine have a similar profile of action. The antiviral effect of these drugs in SARS-CoV-2 infection is mainly to inhibit the virus entry into the host cells. They can also reduce the ACE2 glycosylation, thereby preventing the effective binding of COVID-19 to host cells [11]. Some viruses enter host cells through endocytosis; in the host cell, the virus is transported in endosomes, in which it can replicate [60]. When endosomes bind to the acid lysosome, it breaks the endosome and releases the virus. Chloroquine/hydroxychloroquine has been found to accumulate in lysosomes, interfering with this process [12]. Chloroquine was also shown to increase the endosomal pH, which may interfere with viral entry or exit from host cells [61]. Both drugs also have an immunomodulating effect by inhibiting the production of proinflammatory cytokines, thereby blocking the pathway that leads to the development of acute respiratory distress syndrome (ARDS) [14]. However, recently published studies have not shown any clinical benefits from treatment with these agents [62]. Therefore, both the World Health Organization and the Food and Drug Administration strongly advise against the use of chloroquine and hydroxychloroquine to prevent and/or treat COVID-19 patients, since the risks of their administration outweigh the benefits.

The benefits from colchicine treatment in COVID-19 have also been suggested [63]. For decades, colchicine has been successfully used for the treatment and prevention of gout [64]. In a randomized clinical trial including 105 participants suffering from COVID-19, patients who received colchicine had a statistically significantly longer time to clinical deterioration compared with a control group that did not receive colchicine [65]. Furthermore, in a randomized, double-blinded, placebo-controlled clinical trial, where 72 patients with COVID-19 were randomized to colchicine group (*n* = 36) and control group (*n* = 36), the median time of oxygen supplementation was 4.0 (2.0–6.0) days for the colchicine group and 6.5 (4.0–9.0) days for the placebo group (*p* < 0.001). Median time of hospitalization was 7.0 (5.0–9.0) days for the colchicine group and 9.0 (7.0–12.0) days for the placebo group (*p* = 0.003). To sum up, colchicine reduced the length of oxygen supplementation therapy and hospitalization [64].

Zinc (Zn) is the second most abundant trace metal in the human body, after iron. Previous study suggested that ACE-2 expression is regulated by Sirtuin 1 (SIRT1). Zinc decreases SIRT1 activity, and regulation of SIRT1 by zinc could decrease ACE-2 expression and ultimately viral entry into the cell [66,67]. It has also been reported that Zn specifically inhibits the SARS-CoV RdRp elongation and template binding [68]. More importantly, persistent low serum Zn level has been documented in critically ill patients, which was associated with recurrent sepsis and inversely correlated with sepsis-related mortality, emphasizing the importance of Zn supplementation in COVID-19 therapy [69].

## 4. Pleiotropic Activity of Statins

Statins are highly placed in the international recommendations of cardiac societies due to their impact on reducing the number of coronary events, strokes, and cardiovascular deaths, which has been proven in the results of many clinical trials [70]. Statins are easily available, cheap, and well-tolerated.

Statins have pleiotropic activity. Their main mechanism of action is the reduction of isoprenoids synthesis due to the inhibition of 3-hydroxy-3methylgultaryl-coenzyme A reductase (HMG-CoA), which has a positive effect on vascular endothelium function, and vulnerable atherosclerotic plaque stabilization. They also reduce oxidative stress and have anti-inflammatory and anti-proliferative effects. Statins also have an anticoagulant effect due to the reduction of tissue factor (TF) expression, reduction of thrombin synthesis, enhancement of fibrinolysis, and the inhibition of platelet activation and aggregation [71]. In a randomized controlled trial comparing a four-week rosuvastatin therapy (20 mg once daily) and placebo in patients with prior venous thromboembolism (VT), rosuvastatin substantially improved the coagulation profile, suggesting that statin therapy might be beneficial in patients at risk of recurrent VT [72]. In the population-based registry, the use of statins combined with standard anticoagulation therapy was showen to reduce the number of recurrent pulmonary embolism (PE) episodes by 50% in patient with prior PE [73]. In a murine model, administration of rosuvastatin or atorvastatin improved the efficacy of anticoagulation therapy and reduced the fibrosis of vessels and lungs due to decreased expression of plasminogen activation inhibitor-1 (PAI-1) [74]. In lung fibrosis, impaired balance between urokinase-type plasminogen activator (uPA) and its inhibitor PAI-1 was observed. uPa has been reported to increase the production of prostaglandin E2 by acting as an antifibrinolytic signal leading to remodeling of alveolar epithelium cells [74]. In addition, nonspecific interstitial changes similar to pneumonia and peripheral vasculopathy were observed in mice with uPA receptor deficiency [75]. Hence, statins may restore the impaired balance between pro- and antifibrinolytic molecules by decreasing the concentration of PAI-1 [75].

**Figure 2:**
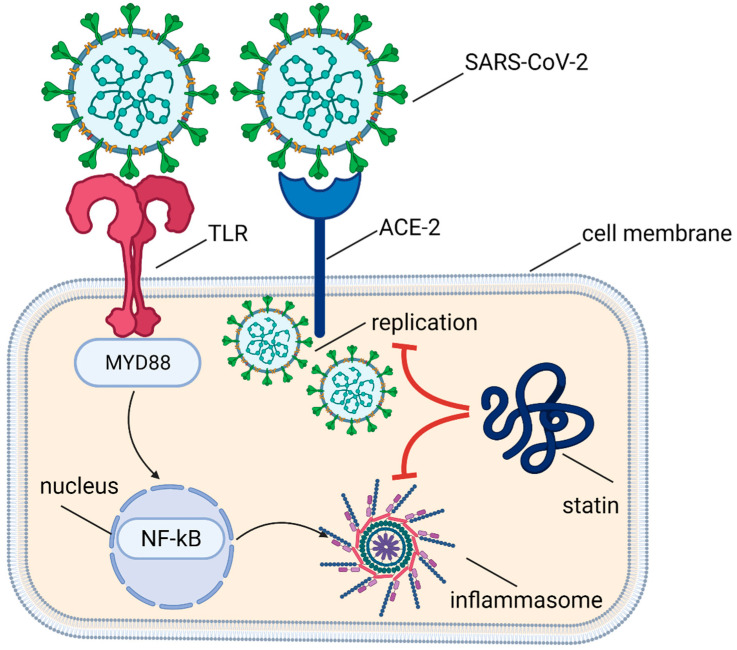
Molecular mechanisms of statins activity in SARS-Cov-2 infection.

Statins also have an immunomodulating effect by inhibiting the expression of the major histocompatibility complex II (MHC II) proteins induced by interferon γ (IFN-γ) in endothelial cells, smooth muscle cells, fibroblasts, and macrophages and by the selective blocking of integrin β2 and adhesive molecule lymphocyte function-associated antigen 1 (LFA-1; CD11a/CD18), which is expressed on leucocytes’ surfaces and binds with intercellular adhesion molecule 1 (ICAM-1) when activated. The immunomodulatory effect of statins is also mediated by their impact on intimal recruitment, differentiation, proliferation, and secretion of immune cells, mainly monocytes/macrophages and lymphocytes T [76]. Many observations to date have also confirmed the effect of statins on the attenuation of the immune response through a decrease in the plasma concentrations of (i) C-reactive protein (CRP), (ii) tumor necrosis factor α (TNF-α), (iii) IL-1 and IL-6, (iv) P-selectin, and (v) activation of TLR-MyD88-NF-κB pathway in endothelial cells, as well as an increase in (i) secretion of anti-inflammatory cytokines and (ii) expression of ACE2 receptor [71,76]. The molecular mechanisms of statins activity in SARS-Cov-2 infection are shown in Figure 2.

Statins also have an antiviral effect through the inhibition of protease activity. The main proteolytic enzyme of SARS-CoV-2 is Mpro. Inhibition of Mpro expression might limit the virus replication in cells. The effect of statins on Mpro expression were compared with antiviral and antiretroviral drugs (favipiravir, nelfinavir, lopinavir) in an in silico computational docking study. Pitavastatin and to a lesser extent rosuvastatin, lovastatin, and fluvastatin were shown to have high binding affinity for Mpro, and thus to be potentially effective Mpro inhibitors. Since it was a computational study, the results required confirmation in clinical trials [77].

In addition, statins have an anti-arrhythmic effect by stabilizing cardiomyocyte cell membrane and positive impact on the action potential. This effect is caused by the attenuation of arrhythmogenic structural remodeling of the myocardium due to up-regulation of GATA-6 transcription factor and inhibition of the Rho pathway, which leads to the reduced dispersion of QT segment [78].

The safety of statins in COVID-19 is also a viable topic. Liver injury, myotoxicity, rhabdomyolysis, and kidney injury are more likely in patients with the acute clinical condition (e.g., severe COVID-19 disease). In addition, most statins are metabolized in the liver by CYP3A4 isoenzyme. Concomitant administration of statin with antiviral agents (e.g., protease inhibitors lopinavir and ritonavir, which are CYP3A4 inhibitors) may potentially increase the risk of statin-related side effects [79]. In such cases, it is recommended to avoid the use of simvastatin and lovastatin, while atorvastatin and rosuvastatin (max. dose of 20 mg daily) can be cautiously combined with antiviral agents [80]. However, the creatine kinase and transaminase levels should be carefully monitored. Apart from lopinavir/ritonavir and atazanavir, statins have not been found to interact with other antiviral drugs used in SARS-Cov-2 treatment. Among statins, rosuvastatin shows the highest safety profile and the lowest interactions with antiviral agents [79].

Altogether, the hitherto available data show that statins can act on more than one molecular target and suggest the benefits from statin use in the treatment of patients with SARS-CoV-2 infection, which may outweigh the risk of potential side effects.

## 5. Statins in Clinical Trials in Respiratory Infections

Due to the postulated anti-inflammatory and immunomodulating effects, statins have been proposed as an adjunctive treatment in viral infections. In animal models, atorvastatin has been shown to reduce the intracellular lipid production and inhibit the replication of the influenza virus [81]. During the influenza H1N1 pandemic in 2009, hospitalized patients with influenza who received statins had less symptoms and reduced overall mortality, compared to those who were not administered statins [82].

Other studies have shown that atorvastatin inhibits the cell entry of hepatitis C virus, Denga virus, West Nile virus, and Japanese encephalitis virus [81]. An in vitro study with Ebola virus showed that treatment with lovastatin reduced the replication of the virus in human macrophages and Huh7 liver cells. The released viruses were less contagious due to the impaired production of envelope glycoproteins [83].

Clinical trials in patients with acute respiratory failure and ARDS show varying effects of statins on clinical outcomes. Studies in mice have shown that statins reduce immune response and lung damage associated with ARDS [84,85]. Secondary analysis of randomized controlled trial data suggested that treatment with simvastatin compared to placebo was associated with improved survival in patients with ARDS [86]. In a randomized trial evaluating the effect of 40 mg atorvastatin daily on the development and course of sepsis among 100 patients in an intensive care unit, the conversion rate to severe sepsis was lower in the atorvastatin group compared to the placebo group (4% vs. 24% *p* = 0.007) [87]. However, because the sample size was relatively small, significant differences between the time of hospitalization and mortality were not found [88]. In contrast, other studies found that the use of rosuvastatin compared to placebo increased 60-day in-hospital mortality in patients with sepsis and ARDS [87] or had no effect on 28-day mortality in ARDS, compared to placebo [89]. In a randomized clinical trial SAILS (Statins for Acutely Injured Lungs from Sepsis), randomization to rosuvastatin had no effect on mortality in patients with sepsis-associated ARDS [90].

Statins can potentially increase the activation of inflammation. Some studies show that prolonged suppression of the TLR-MyD88-NF-κB pathway might result in compensative activation of other innate immune factors, including enhanced production of IL-18 [15]. In the SAILS study, the initially elevated level of IL-18, also caused by the use of statins, increased the 60-day mortality rate [87].

Altogether, some observations regarding the use of statins in acute respiratory failure may discourage the use of these drugs as adjunctive therapy in patients with severe COVID-19 infection who are developing respiratory failure or sepsis. However, in milder forms of viral infections (e.g. influenza virus) statins were shown to have positive immunomodulating and antiviral effects, including a reduction in hospitalization, cardiovascular complications, and deaths [82].

## 6. Statins in SARS-CoV-2 Infection

Most patients suffering from COVID-19 have an increased risk of cardiovascular complications and thromboembolism including deep vein thrombosis or pulmonary embolism or cerebrovascular events, which deteriorate the prognosis.

**Figure 3:**
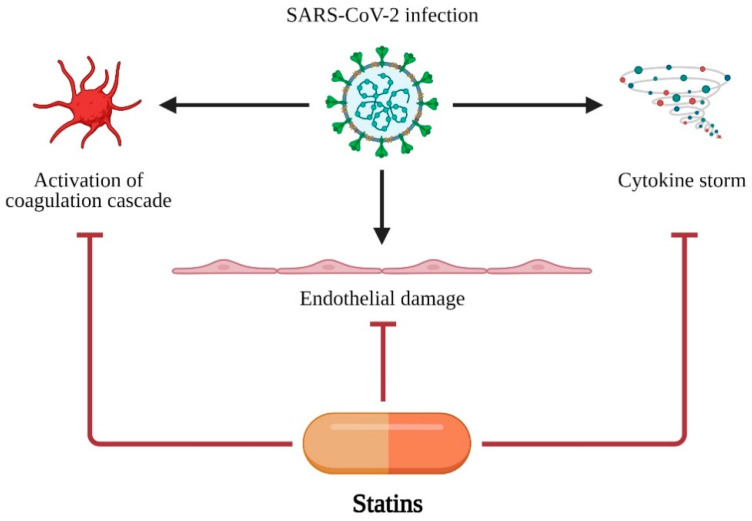
Major mechanisms of statin action in COVID-19 treatment. SARS-CoV-2 infection leads to three major disfunctions: (i) activation of coagulation cascade, (ii) endothelial damage and (iii) cytokine storm. Statins are able to protect organism or at least decrease negative effects of SARS-CoV-2 infection. Created with BioRender.com.

A retrospective analysis of 72,314 COVID-19 cases from China showed that patients with cardiovascular diseases (CVD) presented with significantly higher mortality, compared to those without CVD (10.5% vs. 2.3%) [91]. It has been proposed that statins may have a therapeutic impact on COVID-19 patients through their pleiotropic effects on endothelial function, anti-inflammatory, and antithrombotic effects [92,93,94]. Statins act via three major ways in COVID-19: (i) endothelial protection from damage, (ii) modulation and inhibition of activation of coagulation cascade, and (iii) inhibition of cytokine storm development and progression. The major mechanisms of statin action in COVID-19 are presented in Figure 3.

A retrospective study on 13,981 patients with COVID-19 (1219 receiving statins) showed that statin use was associated with reduced mortality (aHR, 0.58, 95% CI: 0.43–0.80; *p* = 0.001) [95]. In a recent metanalysis, statin use was associated with a 30% risk of death reduction due to COVID-19 [96]. In addition, a 22–30% reduction of the risk of ICU admission was observed in patients on statin therapy. It has been suggested that the positive effect of statins on patient survival may be exerted via modulation of cytokine over-expression, ACE2 expression, and the immune response in COVID-19 patients. Statins also counteract with the cytokine storm, which can occur in severe cases of viral infection such as in SARS-CoV-2 [97,98]. Data regarding the reduced deaths in COVID-19 patients using statins are consistent with findings on the anti-thrombotic, anti-inflammatory, antiviral, and immunomodulatory actions of statins [99,100].

To sum up, statins may be important adjunctive substances in COVID-19 management. However, further data from randomized trials and registries are required to confirm the hitherto observed benefits.

## 7. Conclusions and Future Directions

Inhibitors of HMG-CoA reductase are one of the main groups of drugs used to prevent and treat cardiovascular diseases worldwide. They are widely available, cheap, and well-tolerated. Based on the available data, it can be hypothesized that the use of statins, at least as an adjuvant in antiviral therapy, may be justified. The use of the entire pleiotropic spectrum of statins activity in patients with COVID-19 may lead to the reduction of cardiovascular complications. Due to their beneficial effect on the stabilization of cellular membrane and ion channels in cardiomyocytes involved in the generation of the action potential, statins may reduce the proarrhythmic effect and improve the safety of antiviral therapy. Undoubtedly, the presented topic is very recent and the results of phase 3 of clinical trials are needed to confirm the efficacy and safety of statins in COVID-19 treatment [15]. In each case, the potential benefits and safety of the therapy should be considered individually. It is worth noting that many studies have shown an increased cardiovascular risk in patients withdrawing from statin therapy. According to the guidelines of the European and American Cardiac Societies, in patients with cardiovascular disease or with high cardiovascular risk and concomitant COVID-19 disease, it is recommended to continue statin treatment. However, the initiation of statin therapy de novo in COVID-19 treatment should only be done as part of a clinical trial.

## Data Availability

Not applicable.
